# Faceted Branched Nickel Nanoparticles with Tunable Branch Length for High‐Activity Electrocatalytic Oxidation of Biomass

**DOI:** 10.1002/anie.202005489

**Published:** 2020-07-13

**Authors:** Agus R. Poerwoprajitno, Lucy Gloag, John Watt, Steffen Cychy, Soshan Cheong, Priyank V. Kumar, Tania M. Benedetti, Chen Deng, Kuang‐Hsu Wu, Christopher E. Marjo, Dale L. Huber, Martin Muhler, J. Justin Gooding, Wolfgang Schuhmann, Da‐Wei Wang, Richard D. Tilley

**Affiliations:** ^1^ School of Chemistry The University of New South Wales Sydney NSW 2052 Australia; ^2^ Center for Integrated Nanotechnologies Los Alamos National Laboratory Los Alamos NM 87545 USA; ^3^ Center for Integrated Nanotechnologies Sandia National Laboratories Albuquerque NM 87185 USA; ^4^ Mark Wainwright Analytical Centre The University of New South Wales Sydney NSW 2052 Australia; ^5^ Industrial Chemistry Faculty of Chemistry and Biochemistry Ruhr University Bochum Universitätsstr. 150 44780 Bochum Germany; ^6^ Analytical Chemistry—Center for Electrochemical Sciences (CES) Faculty of Chemistry and Biochemistry Ruhr University Bochum Universitätsstr. 150 44780 Bochum Germany; ^7^ School of Chemical Engineering The University of New South Wales Sydney NSW 2052 Australia; ^8^ Australian Centre for NanoMedicine The University of New South Wales Sydney NSW 2052 Australia

**Keywords:** branched nickel, branching mechanisms, electrocatalysis, HMF oxidation, nanoparticle synthesis

## Abstract

Controlling the formation of nanosized branched nanoparticles with high uniformity is one of the major challenges in synthesizing nanocatalysts with improved activity and stability. Using a cubic‐core hexagonal‐branch mechanism to form highly monodisperse branched nanoparticles, we vary the length of the nickel branches. Lengthening the nickel branches, with their high coverage of active facets, is shown to improve activity for electrocatalytic oxidation of 5‐hydroxymethylfurfural (HMF), as an example for biomass conversion.

## Graphical abstract

**Branched nickel nanoparticles** having controlled branch length are synthesized using a new approach. The branches enable high surface area and surface facets which improve the catalytic activity for biomass electrooxidation.

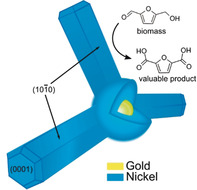

The synthesis of well‐defined 3D nanostructures has been achieved for the fabrication of noble metal nanoparticles.^1^, ^2^, ^3^, ^4^, ^5^ As the focus of energy conversion shifts towards earth‐abundant transition metal catalysts and alkaline electrolytes, approaches are needed to synthesize highly defined and uniform nanoparticles made of first‐row transition metals. A critical feature for uniform nanoparticles is branch dimension and the surface faceting.^5^, ^6^ Ideally for catalysis, nanoparticles should have branch diameters of less than 25 nm to achieve the highest possible surface area.^7^, ^8^ The synthesis also needs to control branch length to enable high exposure of specific active facets.^9^, ^10^, ^11^ This is vital because well‐defined faceting determines the active sites available for catalysis, as has shown to be crucial for branched nanoparticle catalysts.^1^, ^2^, ^12^

Recently, 3D Ni foams with high surface areas have become widely used as high‐performing substrates for a range of electrocatalytic reactions,^13^ including the electrocatalytic oxidation of 5‐hydroxymethylfurfural (HMF).^14^, ^15^ Previous studies have shown high selectivity and high stability of HMF oxidation using 3D porous Ni foam.^14^, ^16^, ^17^ These results suggest that highly uniform branched Ni nanoparticles could show an enhanced catalytic performance for HMF oxidation catalysis due to a large increase in electrochemically active surface area (ECSA) and the faceted branches exposing highly active catalytic sites.

Uniform branched nanoparticles, including semiconductor and metal systems, can be made using face‐centered cubic (*fcc*) cores and hexagonal close packed (*hcp*) crystal structured branches.^1^, ^2^, ^3^, ^4^, ^5^, ^18^ Ni metal typically adopts the *fcc* crystal structure; however, methods for forming nanoscale Ni adopting the metastable *hcp* crystal phase have recently been developed.^19^, ^20^, ^21^ Based on the *hcp* crystal structure of Ni, an opportunity arises for a cubic‐core hexagonal‐branch mechanism to synthesize 3D branched nanoparticles with the uniformity needed for high‐performance catalysis.

Here, we demonstrate growth of *hcp*‐Ni from preformed *fcc*‐Au cores to ultimately obtain branched Ni nanoparticles with an unprecedented degree of control over branch length to form the most uniform 3D Ni nanoparticles reported to date. The faceted branched Ni nanoparticles are more active than nonfaceted and nonbranched Ni nanoparticles. We show that the branch length is a key structural feature for improving the activity of HMF oxidation.

To synthesize uniform branched Ni nanoparticles, it is important to decompose the Ni precursor at a pressure of >3 bar H_2_ gas to ensure that Ni preferentially grows in the metastable *hcp* crystal phase.^20^, ^21^, ^22^, ^23^ A solution of Ni(acac)_2_, hexadecylamine, trioctylphosphine, and mesitylene was added to monodisperse *fcc* Au seeds of 8.7±0.9 nm (Supporting Information (SI), Figure S1) and reacted at 140 °C for 24 h under 5 bar H_2_ in an autoclave_._ The transmission electron microscopy (TEM) image in Figure 1 a shows the successful synthesis of Au‐core Ni‐branched nanoparticles. The nanoparticles self‐assembled to form a chain‐like structure due to their magnetic properties (SI, Figure S2).

**Figure 1:**
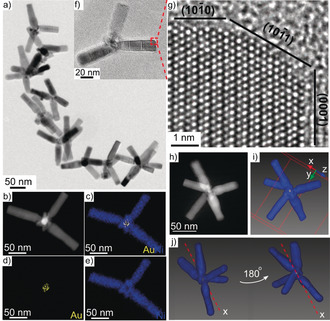
a) TEM image of branched Ni nanoparticles. b) HAADF‐STEM image of a nanoparticle showing contrast between Au core (bright) and Ni branch (dark). c–e) STEM‐EDX maps of Au/Ni, Au, and Ni. f) TEM image of an individual branched nanoparticle. g) HRTEM image of a branch indicated by the red box in (f). h,i) STEM image and surface‐rendered visualization of a nanoparticle with five branches. The choice of axis labeling is arbitrary. j) Visualization of the reconstructed model rotated 180° about the *x* axis.

The synthesis forms nanoparticles with well‐defined branches that are uniform in size and shape. The branches have lengths of 70±10 nm and widths of 20±2 nm with over 80 % of the nanoparticles having between two and four branches (Figure S3, SI). The branches are decorated with a 3 nm oxide layer (Figure S4, SI). The Ni branches are attached to the Au cores via a 8±2 nm thick Ni shell (measured from the edge of the Au core to start of the branch), as evidenced by the distinct bright Au core and darker Ni branch and shell regions in the high‐angle annular dark‐field scanning TEM (HAADF‐STEM) and energy‐dispersive X‐ray spectroscopy mapping analysis (STEM‐EDX) in Figure 1 b–e and Figure S5 (SI), indicating that Au is not exposed to the surface. X‐ray diffraction patterns (XRD) and selected area diffraction patterns confirm the presence of *fcc*‐Au, *fcc*‐Ni, and *hcp*‐Ni (SI, Figure S6).The Ni branches remain defined and attached to the core with no observable degradation of the nanoparticles over months when stored in toluene (SI, Figure S7).

The surface facets of the branches were analyzed by aberration‐corrected high‐resolution TEM (HRTEM) as shown in Figure 1 f,g and Figure S8 (SI). The branches have a well‐ordered atomic arrangement which can be indexed by a regular *hcp* crystal structure that extends along the *c*‐axis (SI, Figure S9). The surface facets are {10$\bar{1}$ 0} facets on the side, {10$\bar{1}$ 1} and {0001} on the tip. The extended branch along the *c*‐axis results in high proportion of {10$\bar{1}$ 0} facets as compared to {10$\bar{1}$ 1} and {0001} facets.^9^

The 3D morphology of the branches was analyzed by STEM tomography (SI, Movie S1). The branches grow without preferential direction with respect to the core, as shown by STEM (Figure 1 h) and tomography (Figure 1 i,j). The tip of the branches has a hexagonal shape (SI, Figure S10). A time‐dependent study (SI, Figure S11) revealed the three‐step growth mechanism of branched Ni nanoparticles: 1) heterogeneous nucleation to form core–shell Au–Ni nanoparticles, 2) crystallization of the amorphous shell, and 3) growth into *hcp* Ni branches. The large lattice mismatch between *fcc*‐Au (111) and *hcp*‐Ni (0001) prevents epitaxial growth of Ni branches onto the Au seed. The Ni shell offsets the strain between these highly mismatched planes similar to the Au–Ru system.^1^

Control of the branch length is achieved by varying the concentration ratio of Ni(acac)_2_ to Au seeds as shown in Figure 2 a and Figure S12 (SI). The branch length increased from 70±10 nm (25:1 Ni/Au) to 108±18 nm (37.5:1 Ni/Au) and 155±22 nm (50:1 Ni/Au). The concentration ratio needs to be below 75:1 Ni/Au to avoid formation of Ni monometallic nanoparticles (SI, Figure S13). Above a ratio of 75:1 Ni/Au, the Ni concentration exceeds the critical concentration for homogeneous nucleation as shown by the presence of Ni urchin‐like structure.^24^ In addition, the concentration ratio needs to be above 12.5:1 Ni/Au in order to ensure that enough Ni is available for growth of branches. At low concentration ratios (12.5:1 Ni/Au), both core–shell structures and short branches are formed.

**Figure 2:**
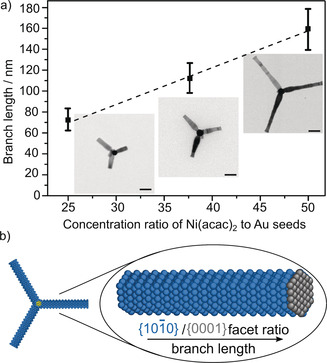
a) Branch length (nm) versus concentration ratio of Ni(acac)_2_ to Au seeds with representative TEM images of each sample. Error bars shows standard size deviation. Scale bars are 50 nm. b) A model showing the surface faceting along the branch. Blue and gray atoms represent Ni atoms on {10$\bar{1}$ 0} facets and {0001} facets, respectively. The longer branch results in higher proportion of {10$\bar{1}$ 0} facets compared to {0001} facets.

At a higher Ni/Au ratio, there are relatively small changes in the width of the nanoparticle branches: an increase by 3 nm to 23 nm and the average number of branches with an increase from three to four. This indicates the majority of the growth is along the *c*‐axis making longer branches. This growth mechanism can be due to the strong binding of amine surfactant to {10$\bar{1}$ 0} facets or kinetically preferred growth along the *c*‐axis.^25^, ^26^ The above set of experiments demonstrates the high level of control achieved in this synthesis strategy, with tunable branch lengths while maintaining branch widths below 25 nm.

The ability to control the branch length without altering the width of branches enables tuning of the ratio of {10$\bar{1}$ 0} facets on the sides and {0001} on the tips (Figure 2 b). {10$\bar{1}$ 0}/{0001} facet ratios of 18, 25, and 37 were calculated for nanoparticles with 70 nm, 108 nm, and 155 nm branches, respectively (SI, Table S1). The {10$\bar{1}$ 0} and {0001} facets have both shown different catalytic activities and stabilities for many reactions.^9^ The tuneable facet ratio allows us to identify the more active facet for HMF electrooxidation. This is significant because unlike an isotropic nanoparticle in which the effect of faceting can be studied by comparing different shapes,^11^ the effect of faceting in an anisotropic branched nanoparticle can only be studied by tuning the branch length, which has not been achieved with such uniformity for Ni. Our approach successfully produces uniform nanosized branched Ni nanoparticles with a high level of control over branch dimensions compared to previously reported branched Ni nanoparticles synthesized by kinetic growth and polymorphism.^21^, ^22^, ^27^, ^28^ A combination of seeded growth and high‐pressure hydrogen gas is the key to achieve this control.

Au seeds are needed to ensure uniformity, as evident from a similar synthesis in the absence of Au seeds, which resulted in randomly branched Ni nanoparticles that were polydisperse size and shape (SI, Figure S15). High pressure is needed to produce *hcp* branches, as shown by a similar synthesis with lower pressure H_2_ (1 bar) which produced *fcc* Ni nanocubes with no branches.^29^ This high degree of control is similar to that applied for the formation of branched CdSe, CdTe, or PdRu nanoparticles synthesized by switching crystal structure.^2^, ^5^, ^30^ By controlling the synthesis of branched Ni nanoparticles, we achieve both 3D structure and controlled surface facets which are key for highly active oxidation electrocatalysis.

The catalytic performance of the branched Ni nanoparticles for the electrochemical oxidation of HMF was evaluated in 0.1 m KOH containing 10 mm HMF as the electrolyte. At pH 14, HMF degrades to humin‐type products decreasing the HMF concentration (SI, Figure S16).^15^ The measured catalytic current was normalized by ECSA (SI, Figure S17). The catalytic activity of Ni nanoparticles with 70 nm branches at 1.5 V is 3× higher (Figure 3 a) than for nonfaceted, nonbranched amorphous Ni sphere nanoparticles (SI, Figure S18). The linear sweep voltammograms (LSV) of both Ni spheres and branched Ni nanoparticles in the presence of HMF show two oxidation peaks. For the branched Ni nanoparticles, the onset of HMF oxidation around 1.43 V coincides with the oxidation of Ni^2+^ to Ni^3+^ (SI, Figure S19), suggesting that the formation of Ni^3+^ is the limiting process.^31^, ^32^ The higher activity of branched Ni nanoparticles as compared with nonbranched spherical Ni nanoparticles is thus attributed to the 40 mV lower overpotential required to oxidize Ni^2+^ to the active Ni^3+^ as shown by LSV in the absence of HMF (SI, Figure S20). HMF oxidation reaches a diffusion‐controlled regime at around 1.55 V concurrently with the progressing oxygen evolution reaction (OER), as shown by the increasing current density at potentials above 1.58 V. The higher overpotential of OER required to achieve the same current density (SI, Figure S19) shows that HMF oxidation is more favorable than OER and can be an alternative anodic reaction for water‐splitting cells.^14^

**Figure 3:**
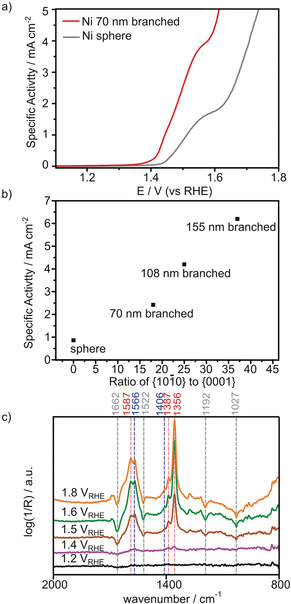
a) LSV of branched Ni nanoparticles (red line) and amorphous Ni spheres (gray line) in 0.1 m KOH containing 10 mm HMF. b) Specific activity versus ratio of {10$\bar{1}$ 0} to {0001} facets showing the activity increases proportionally with the increasing ratio of exposed of {10$\bar{1}$ 0} facets. c) Operando ATR‐FTIR spectra recorded after 10 min of various applied potentials between 1.2 to 1.8 V vs. RHE in 0.1 m KOH and 10 mm HMF.

To evaluate the electron transfer kinetics of HMF oxidation at the nanoparticles, electrochemical impedance spectroscopy (EIS) measurements were conducted. Figure S21 (SI) shows that the charge transfer resistance of branched Ni nanoparticles is 56 Ω, which is lower than that of the amorphous Ni sphere nanoparticles (161 Ω) at 1.5 V. The faster charge transfer at the *hcp* crystal phase structure is likely the reason for the higher activity for HMF oxidation.^33^ The Tafel plots (SI, Figure S22) show slopes of 52.6 mV dec^−1^ and 80.9 mV dec^−1^ for branched Ni and Ni sphere nanoparticles, respectively. The lower Tafel slope supports a faster charge transfer rate for the branched Ni nanoparticles.^32^, ^34^

The control of the branch length further improves the electrocatalytic activity as shown in Figure 3 b and Figure S23 (SI). XRD shows that the crystal structure is consistent across all samples with *fcc*‐Ni and *hcp*‐Ni and *fcc*‐Au present (SI, Figure S24). X‐ray photoelectron spectroscopy (XPS) shows that the surface Ni^2+^ oxidation state is maintained as the Ni branches lengthen (SI, Figure S24). This indicates that the high activity of 155 nm branched Ni nanoparticles is not due to changes in surface oxidation or crystal structure. The branch length directly controls the ratio of {10$\bar{1}$ 0} facets to {0001} facets, as calculated by the number of atoms (SI, Table S1). The specific activity for HMF oxidation on Ni branches increases proportionally with the increasing ratio of {10$\bar{1}$ 0} facets to {0001} facets. Increasing the ratio of {10$\bar{1}$ 0}/{0001} by 1.4× (108 nm branches) and 2.1× (155 nm branches) resulted in 1.8× and 2.5× higher specific activity than that at the 70 nm branches, respectively. In comparison to Ni spheres, the 155 nm branched Ni nanoparticles exhibited ≈7.2× higher specific activity. This specific activity of the branched Ni nanoparticles is almost 4 times higher than that from previous report (Table S2, SI). This facet ratio to activity correlation suggests that the {10$\bar{1}$ 0} facet is more active for HMF oxidation than the {0001} facet. Previous density functional theory (DFT) calculations have noted that the energetics of O−H bond dissociation of HMF could be used to assess its oxidation activity.^35^ Our DFT calculations showed that HMF adsorption is more favorable on the Ni {10$\bar{1}$ 0} facet (−1.05 eV) than on the {0001} facet (−0.97 eV). Further, they also revealed that O–H dissociation is more favorable on the Ni {10$\bar{1}$ 0} facet (−0.51 eV) than on the {0001} facet (−0.37 eV) (SI, Figures S25 and S26). Our results show the importance of synthetically controlling the length of the branches to increase the exposure of more active {10$\bar{1}$ 0} facets which is vital for improved activity in HMF electrooxidation.

Operando electrochemistry‐coupled attenuated total reflection infrared (EC‐ATR‐IR) spectroscopy at various potentials was conducted to confirm the conversion of HMF (Figure 3 c). At 1.5 V, HMF bands at 1027, 1192, 1522, and 1662 cm^−1^ (gray lines) were depleted and the bands associated with 5‐formyl‐2‐furancarboxylic acid (FFCA) and 2,5‐furandicarboxylic acid (FDCA) were observed at 1356, 1387, 1406, 1566 cm^−1^, and 1587 cm^−1^ (blue and red lines), confirming that the oxidation peak at 1.5 V in Figure 3 a originates from HMF oxidation. At 1.4 V, 5‐hydroxymethyl‐2‐furancarboxylic acid (HMFCA) bands were observed at 1386 and 1361 cm^−1^ (Figure 3 c). Therefore, we can say that the HMF oxidation occur via the HMFCA pathway which is consistent with previous reports on Ni‐based catalysts.^15^, ^32^ The bands of the products increase in intensity at more positive potentials up to 1.8 V, which indicates that HMF oxidation is still occurring under OER conditions. This result demonstrates the effective conversion of HMF to FDCA. Post‐catalysis TEM characterization performed after an hour of chronoamperometry shows that the morphology and surface faceting is stable (SI, Figure S27), with 1.7 times more of the active Ni^3+^ observed in the surface oxidation states (SI, Figure S28).

In conclusion, we demonstrate that the cubic‐core hexagonal‐branch growth mechanism enables the formation of branched Ni nanoparticles with tunable branch length which are important for catalytic applications. The key of this synthesis is the combination of seeded‐growth and high hydrogen pressure. The branched Ni nanoparticles have higher activity in anodic HMF oxidation than amorphous Ni sphere nanoparticles. This high performance is attributed to high surface area and exposure of {10$\bar{1}$ 0} facets along the branches. This concept opens up the opportunity for the synthesis of various well‐defined branched nanoparticles with bimetallic structure for different electrocatalytic applications.

## Conflict of interest

The authors declare no conflict of interest.

## Supporting information

As a service to our authors and readers, this journal provides supporting information supplied by the authors. Such materials are peer reviewed and may be re‐organized for online delivery, but are not copy‐edited or typeset. Technical support issues arising from supporting information (other than missing files) should be addressed to the authors.

Supplementary

Supplementary
