# Supplementary material for: Faceted Branched Nickel Nanoparticles with Tunable Branch Length for High‐Activity Electrocatalytic Oxidation of Biomass
Source: Angew Chem Int Ed Engl. 2020 Jul 13;59(36):15487–91. doi: 10.1002/anie.202005489 (PMC7497201; doi:10.1002/anie.202005489)
Supplement: Supplementary file 1 — Supplementary [file ANIE-59-15487-s001.pdf]

Angewandte Chemie
Eine Zeitschrift der Gesellschaft Deutscher Chemiker

Supporting Information

# Faceted Branched Nickel Nanoparticles with Tunable Branch Length for High-Activity Electrocatalytic Oxidation of Biomass

*Agus R. Poerwoprajitno, Lucy Gloag, John Watt, Steffen Cychy, Soshan Cheong, Priyank V. Kumar, Tania M. Benedetti, Chen Deng, Kuang-Hsu Wu, Christopher E. Marjo, Dale L. Huber, Martin Muhler, J. Justin Gooding,* Wolfgang Schuhmann,* Da-Wei Wang,* and Richard D. Tilley**

anie_202005489_sm_miscellaneous_information.pdf
anie_202005489_sm_movie_Ni_branch.mp4

# SUPPORTING INFORMATION

**Table of Contents**

**Experimental details**
**Figure S1** | TEM image of Au nanoparticles used as seeds for branched Ni nanoparticles.
**Figure S2** | Magnetization curves of branched Ni nanoparticles.
**Figure S3** | Statistical analysis of branch length, branch width and number of branches.
**Figure S4** | EDS mapping of the oxide layer.
**Figure S5** | EDS mapping of branched Ni nanoparticles.
**Figure S6** | XRD and SAED pattern of branched Ni nanoparticles.
**Figure S7** | TEM image of branched Ni nanoparticles after 8 months.
**Figure S8** | Crystallography and surface facet study of branched Ni nanoparticles by HRTEM.
**Figure S9** | HRTEM and corresponding FFT of different areas.
**Figure S10** | TEM images of a nanoparticle with 4 branches.
**Figure S11** | Growth study of intermediate products.
**Figure S12** | TEM images of branched Ni nanoparticles with different branch length.
**Figure S13** | TEM images of branched Ni nanoparticles with high and low Ni:Au ratio.
**Figure S14** | Statistical analysis of branch width and number of branches of all nanoparticles. **Figure S15** | TEM image of branched Ni nanoparticles without Au seeds.
**Figure S16** | Chronoamperometry profile at pH 14.
**Figure S17** | Electrochemical active surface areas (ECSAs) analysis of branched Ni nanoparticles.
**Figure S18** | TEM image of amorphous sphere Ni nanoparticles.
**Figure S19 |** Linier sweep voltammetry (LSV) of branched Ni nanoparticles with and without HMF.
**Figure S20** | Comparison of oxidation scan of branched and sphere Ni nanoparticles.
**Figure S21** | Electrochemical impedance spectroscopy (EIS) of branched and sphere Ni nanoparticles.
**Figure S22** | Tafel slope analysis of branched and sphere Ni nanoparticles.
**Figure S23** | LSV curves of branched Ni nanoparticles with different branch length.
**Figure S24** | XRD and XPS analysis of branched Ni nanoparticles with different branch length.
**Figure S25** | Structural models used in DFT modelling.
**Figure S26** | Potential energy diagram of O-H bond dissociation on Ni (0001) and (10-10) facets.
**Figure S27** | Post-catalysis TEM characterization.
**Figure S28** | XPS spectra of branched Ni nanoparticles before and after catalysis.
**Calculation of facet ratio**
**Table S1 |** Calculation of facet ratio of branched Ni nanoparticles with different branch length. **Table S2 |** Comparison of HMF oxidation activity in alkaline solution.
**Movie S1** | Series of HAAD-STEM images of a branched Ni nanoparticle at different tilting angles.

## Experimental details

**Synthesis of 8 nm Au nanoparticles.** 0.08 g gold (III) chloride trihydrate (Aldrich, 99%) and 0.74 g dodecylamine (Aldrich, 99%) was dissolved in 4 mL toluene (chem-supply, 99%). The solution was then transferred to a Fisher Porter bottle fitted with a bivalve. The bottle was evacuated of air and purged with nitrogen before filling with 3 bar hydrogen gas and placing in oil bath at 60°C. After 24 h, the bottle was cooled down and the hydrogen was released. The deep purple solution washed once in a centrifuge for 15 mins at 5000 rpm using a 1:1 mixture of toluene: ethanol. The nanoparticles were stored in 10 mL mesitylene (Aldrich, 98%).

**Synthesis of branched Ni nanoparticles.** 0.08 g nickel (II) acetylacetonate (Aldrich, 95%), 0.0024 g Au seeds, 1.4 g hexadecylamine (Aldrich, 98%) and 70 µL Tryoctylphosphine (Aldrich, 97%) was dissolved in 20 mL mesitylene. The ratio between Au seed and Ni precursor can be tuned to control the branch length. The solution was evacuated and flushed with argon and then transferred into an autoclave (non-stirred pressure vessel 4766 from Parr instrument company) using gas-tight Hamilton syringe. The reactor was filled with 5 bar hydrogen gas and placed in oil bath at 140°C. After 24 hours, the reactor was cooled down and the hydrogen was released. The nanoparticles were separated by magnetic force and redissolved in toluene. Nanoparticles was loaded on carbon Vulcan XC-72 for electrochemical measurement.

**Characterization.** Transmission electron microscope (TEM) samples were prepared by drop-casting a solution of nanoparticles suspended in cyclohexane onto a carbon coated copper grid or a grid containing a 10 nm thick SiN window. Low resolution TEM images were taken on FEI Tecnai G2 20 TEM operating at 200 kV and high-resolution TEM (HRTEM) images were taken on JEOL JEM-F200 microscope at 200 kV. The HRTEM image in Figure 1 was taken on FEI Titan ETEM with Image $C_s$ corrector operating at 300 kV. STEM images and EDX maps were recorded using a JEOL F200 microscope. Size distribution was analyzed from at least 200 nanoparticles using ImageJ software. STEM images for tomographic reconstruction were acquired on the same JEOL microscope over a tilt range of -71° to +61° at 2° increment using a JEOL high tilt sample stage. Image recording, reconstruction and visualization were performed using the TEMography software package. 3D reconstruction was achieved via a SIRT (simultaneous iterative reconstruction technique) algorithm and visualization by isosurface rendering of the tomographic data cube.

X-ray diffraction (XRD) patterns were performed using an MPD (PANalytical) Xpert Multipurpose X-ray diffraction system with a Cu Kα source. X-ray photoelectron spectroscopy (XPS) was performed using an ESCALAB250Xi (Thermo Scientific, UK) using a monochromated Al Kalpha source (1486.68 eV) operating at 120W (13.8 kV x 8.7 mA) with an analysis spot size of 500 micrometres and a take-off angle of 90 degrees. The pass energy was 100 eV for survey scans and 20 eV for regional scans. Data was processed using the Avantage software package (Thermo Scientific, UK) and binding energy referenced to the C1s peak at 284.5 eV.

**Magnetic measurement.** DC magnetization curves were recorded from -2.38 x $10^3$ kA/m to 2.38 x $10^3$ kA/m at 293 K using a Quantum Designs Versalab vibrating sample magnetometer (VSM).

**Electrochemical measurement.** The electrochemical measurements were carried out in a three-electrode system using 0.1 M KOH containing 10 mM HMF as the electrolyte with Pt mesh as counter electrode and 1 M Hg|HgO|NaOH as reference electrode. The catalyst ink was prepared as 5.0 mg $mL^{-1}$ dispersion in 49:49:2 vol.% water, ethanol, Nafion®. In a typical experiment, 5 µL of the catalyst ink was deposited on a glassy carbon surface (0.07 $cm^{-2}$) as the working electrode. The final catalyst loading was 0.35 mg $cm^{-2}_{geom}$ with 26%~32% Ni as determined by ICP. The electrocatalytic of HMF oxidation was obtained by linier sweep voltammetry at 5 mV $s^{-1}$.

All potentials are given relative to the reversible hydrogen electrode (RHE) according to equation:

$$E_{RHE} = E_{Hg/HgO} + 0.059 \,.\, pH + E^0_{Hg/HgO}$$

The electrochemically active surface areas (ECSA) were calculated from the integration of $Ni^{2+/3+}$oxidation peak in 0.1 M KOH.[1] An exponential baseline was subtracted.

The electrochemical impedance spectroscopy (EIS) was performed with a Solartron SI 1287 electrochemical interface and SI 1260 impedance/gain-phase analyzer. The EIS test was conducted with frequency range from $10^5$ to $10^{-1}$ with AC amplitude of 10 mV.

**Operando electrochemistry coupled attenuated total reflection infrared spectroscopy.** The catalyst ink was prepared as 5.0 mg $mL^{-1}$ dispersion in 49:49:2 vol.% water, ethanol, Nafion®. This catalyst ink was carefully dropped on a glassy carbon ring of a homemade bore hole electrode (BHE) (0.446 $cm^2$) as the working electrode to obtain a loading of 210 µg $cm^{-2}$ and was allowed to dry for 1 hour. Pt mesh and Ag|AgCl|3M KCl were used as counter and reference electrode, respectively. The potential cycling was conducted in 0.1 M between 1.0 to 1.6 V vs RHE with a scan rate of 100 mV $s^{-1}$ until stable voltammograms were achieved, here 20 cycles. The electrode was then transferred to spectroelectrochemical setup and immersed in 10 mM HMF and 0.1 M KOH electrolyte (Bruker Tensor 27, A530/P reflection unit modified with a homemade cell geometry). Mid-IR radiation was emitted by a globar, modulated via an interferometer and detected in a liquid $N_2$-cooled MCT (mercury cadmium telluride) detector. The optical bench and the sample compartment were constantly purged with dried air. All potentials were applied by a PalmSens $EmStat^3$ 4 WE using the software Multitrace 4.

The distance between the electrode and the Ge internal reflection element was 20 µm and a flow rate of 5 µL $min^{-1}$ was induced by a Medorex peristaltic pump with 3 rolls. A reference spectrum was recorded at the open circuit potential (OCP) averaging 200 IR scans with a resolution of 4 $cm^{-1}$. Afterwards, a potential program was applied i.e. a potential of 1.2 to 1.8 V vs RHE was applied in steps of 0.1 V for 10 min each. Between each step, the potential was decreased to 1.0 V vs RHE for 4 min. IR spectra were recorded every 2 min averaged over 200 IR scans (1.5 min, 4 $cm^{-1}$ resolution). The IR spectrometer was controlled by the OPUS 7.2 software. Reference spectra were recorded in 10 mL 0.1 M Ar saturated KOH and 5 mM Ar-saturated solution of the respective substance.

**DFT calculations**. The VASP code was used for DFT calculations.[3,4] The core electrons were treated using the projector augmented wave method (PAW) method,[5] and the Perdew-Burke-Ernzerhof (PBE) exchange-correlation functional[6] was employed. We used a 12.6 Å × 10.74

Å unit cell containing 100 Ni atoms to model Ni (0001) surface, while a 10.74 Å × 12.26 Å unit cell containing 120 Ni atoms was used to model Ni (10-10) surface. The kinetic energy cut-off was set to 500 eV and a gamma-point k-grid was used. All relaxations proceeded until the residual forces on atoms were less than 0.03 eV/Å. A vacuum region greater than 12 Å was used to avoid interactions between neighboring slabs.

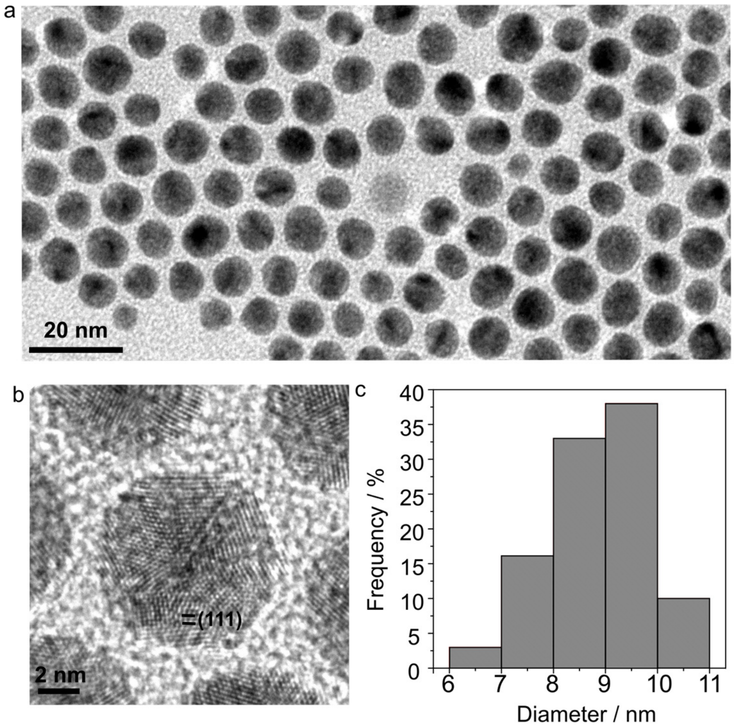


**Figure S1. a)** TEM image of Au nanoparticles. **b)** HRTEM image of an icosahedral Au nanoparticle. **c)** Size distribution of Au nanoparticles with diameters of 8.7 ± 0.9 nm.

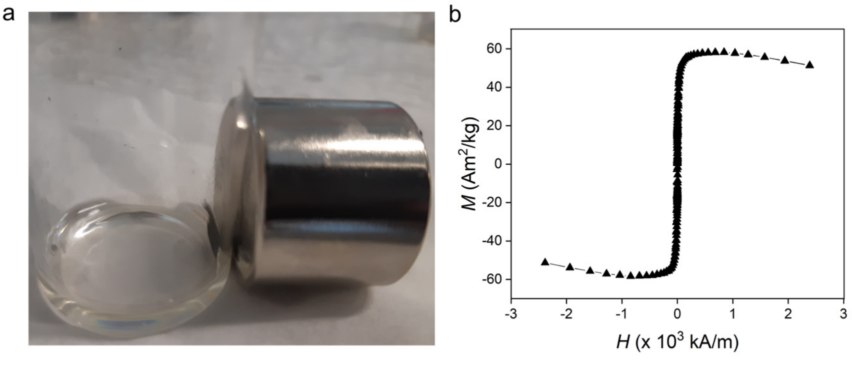


**Figure S2**. **a)** Separation of branched Ni nanoparticles by magnet. **b)** Magnetization curves of 293 K of branched Ni nanoparticles. The branched Ni had a magnetic saturation of 56 $Am^2/kg$ of Ni which is similar to bulk Ni and previous branched Ni nanoparticles.[7-9]

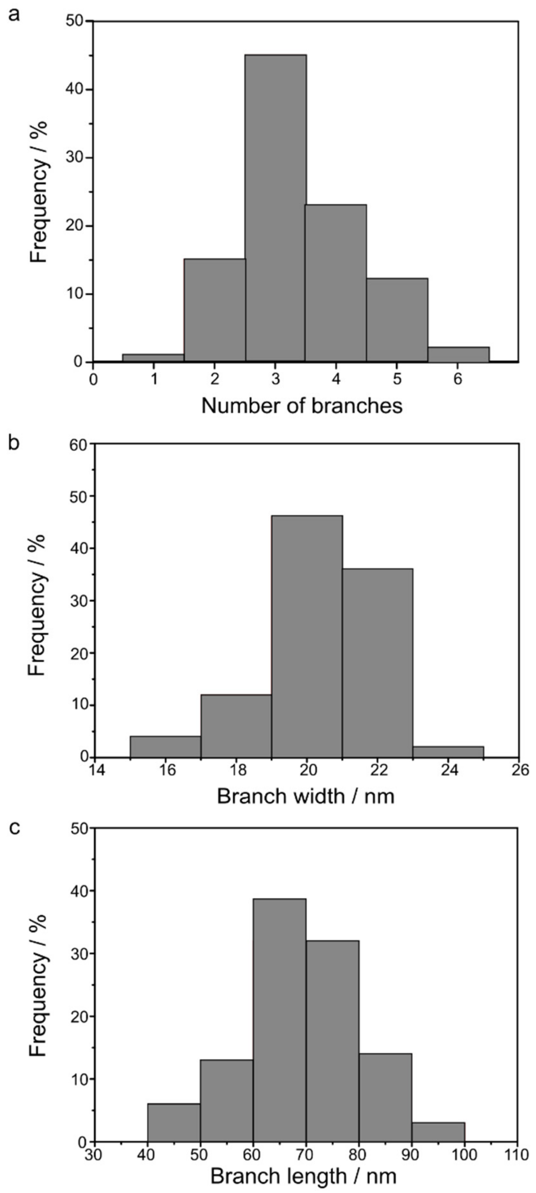


**Figure S3.** Statistical analysis of **a)** number of branches, **b)** branch width and **c)** branch length of branched Ni nanoparticles with concentration ratio of 25:1 Ni:Au. The number of branches per particle varies between 1 and 6, with 82% having either more than 2 branches. The branch width is 20 ± 2 nm. The length of the branches is 70 ± 10 nm. The branch length is measured from the edge of the core to the tip of the branch. The number of branches was analyzed at different tilting angles. The histograms were obtained from counting 200 nanoparticles.

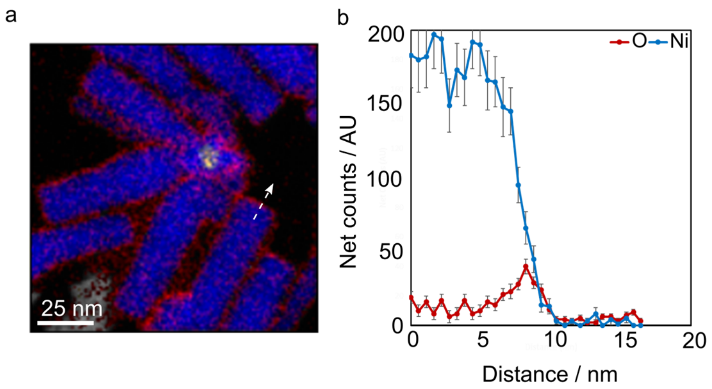


**Figure S4. a)** EDX mapping of Au (yellow), Ni (blue) and O (red) in a branched Ni nanoparticle and **b)** line scan across the area shown by the arrow in (a). Ni branches have approximately 3 nm Ni oxide.

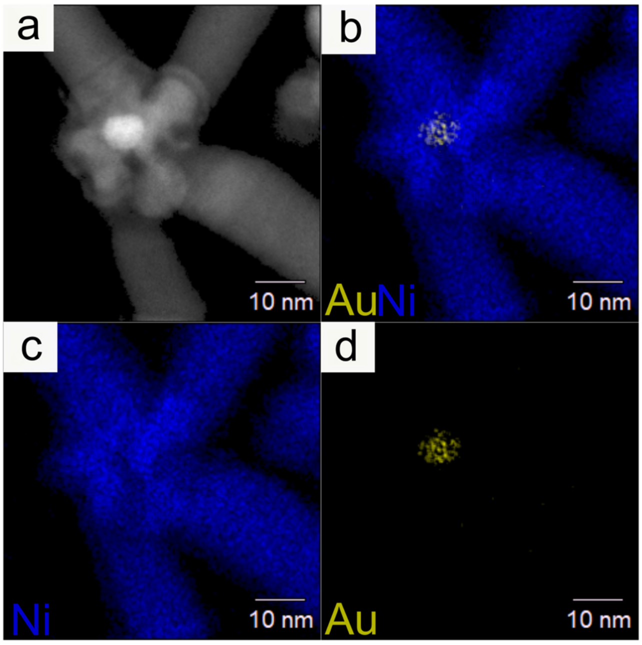


**Figure S5.** EDS mapping of branched Ni nanoparticles showing that Au core is covered by Ni shell.

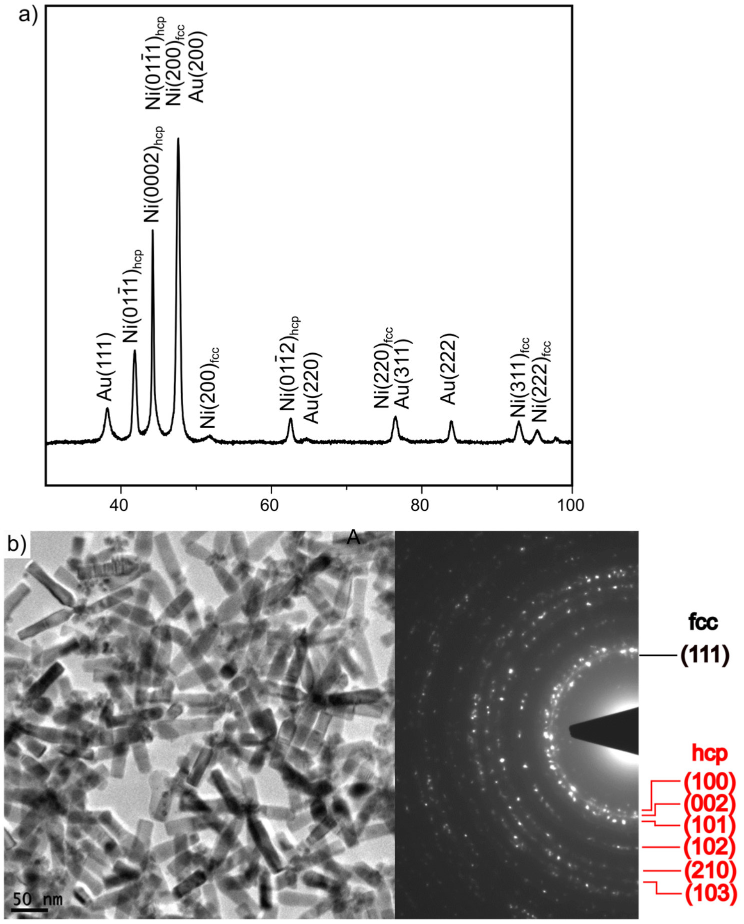


**Figure S6. a)** XRD and **b)** SAED pattern of branched Ni nanoaprticles showing the presence of *fcc*-Au, *fcc*-Ni and *hcp*-Ni

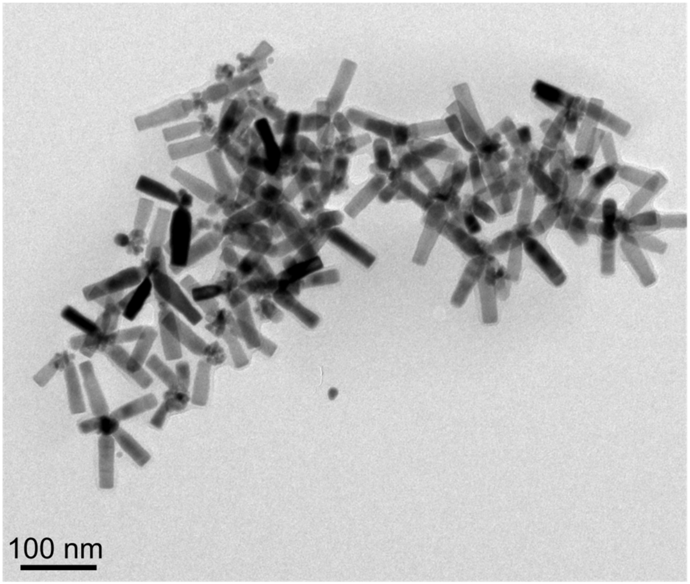


**Figure 7.** TEM image of branched Ni nanoparticles after storage for 8 months dispersed in toluene, demonstrating the nanoparticle morphology is stable over time.

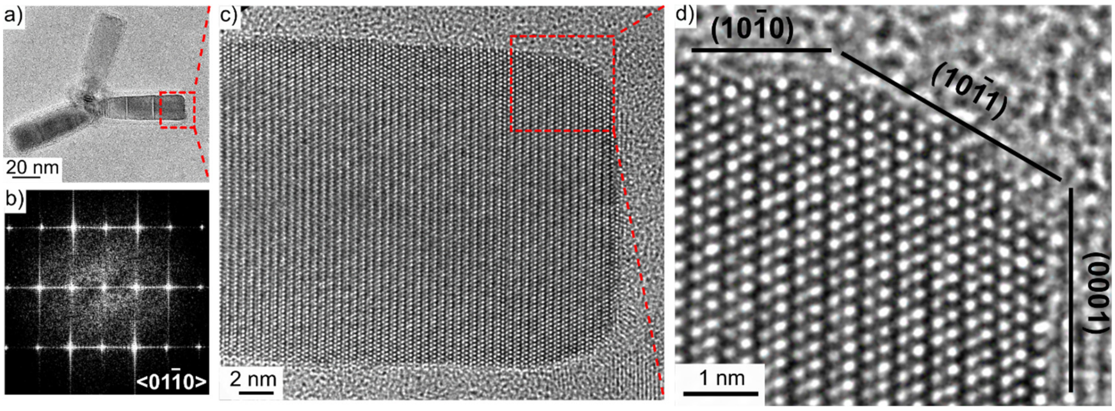


**Figure S8. a)** TEM image of a single nanoparticle. **b, c)** Fast Fourier transform (FFT) and HRTEM image of the red box in (a). FFT can be indexed to *hcp* crystal phase **d)** Atomic-resolution image of the red box in (c) showing the surface facets.

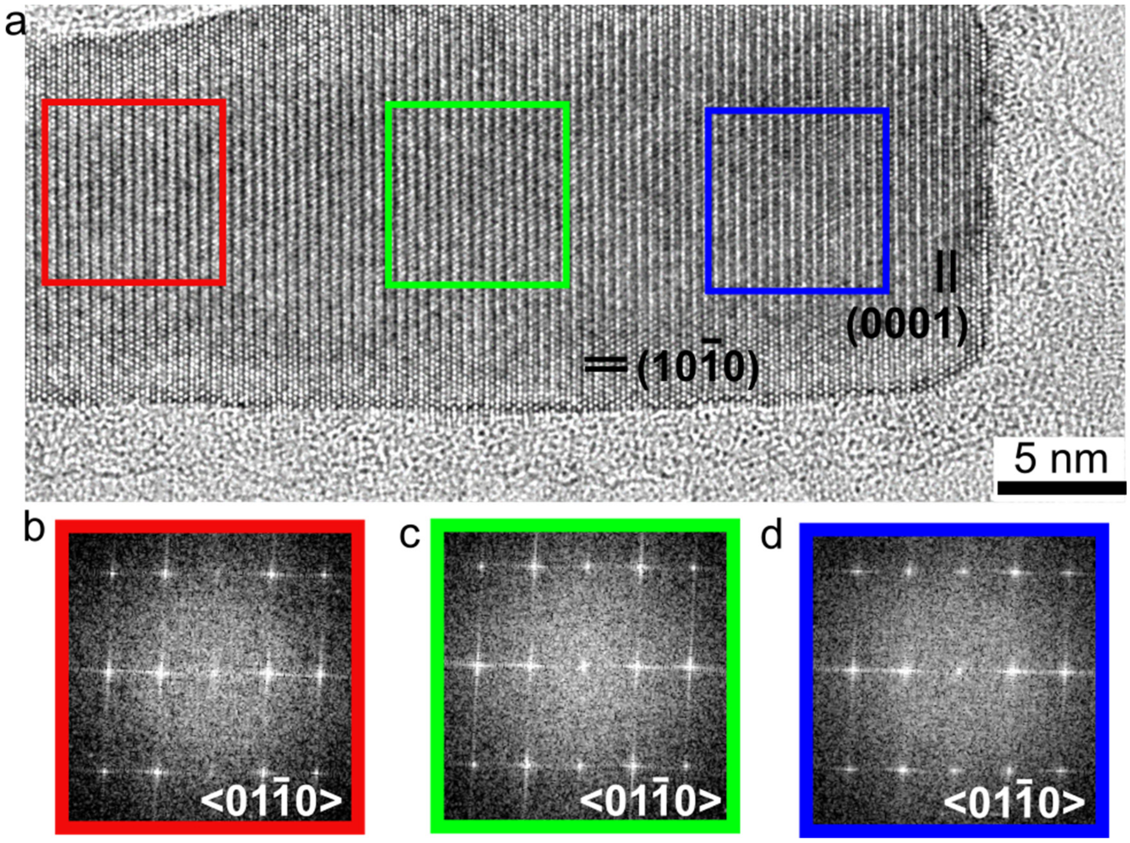


**Figure S9. a)** HRTEM image of a branch. **b-d)** The corresponding FFT of the different areas in image (a) that matches a hcp crystal structure showing that *hcp* crystal structure is formed along the branches.

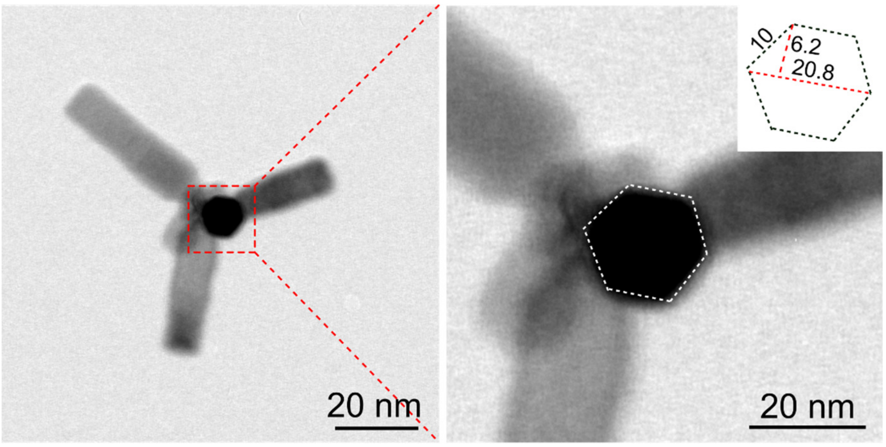


**Figure S10.** TEM images of a nanoparticle with 4 branches confirming that the Ni branches have a hexagonal shape at the tip.

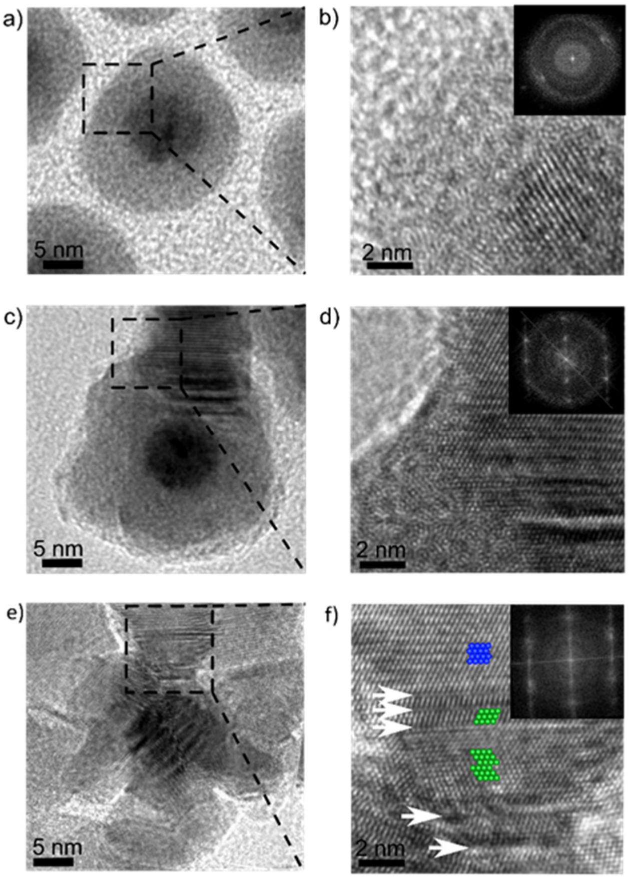


**Figure S11**. **a)** TEM image of an Au-Ni core-shell nanoparticle isolated after 4 h. **b)** HRTEM and FFT (inset) of the core-shell interface from the black box in (a). **c)** TEM image of an Ni nanoparticle with a single branch isolated after 12 h. **d)** HRTEM and FFT (inset) of the shell and branch interface from the black box in (c). **e)** TEM image of a branched Ni nanoparticle isolated after 24 h. **f)** HRTEM and FFT (inset) of the shell-branch interface from the black box in (f).

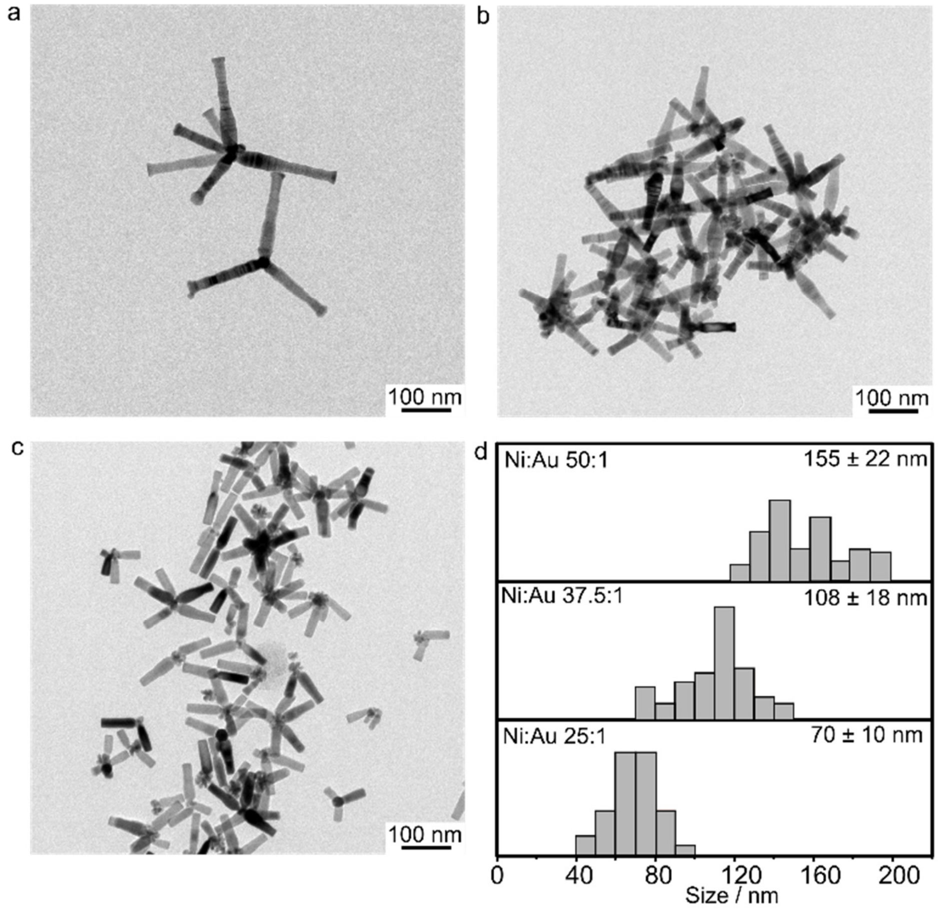


**Figure S12. a-c)** TEM images of branched Ni nanoparticles synthesized with different Ni:Au ratio a) 50:1 b) 37.5:1 c)25:1. **d)** Size distribution of Ni nanoparticles with different branch length while the branch width remains the same (20-23 nm).

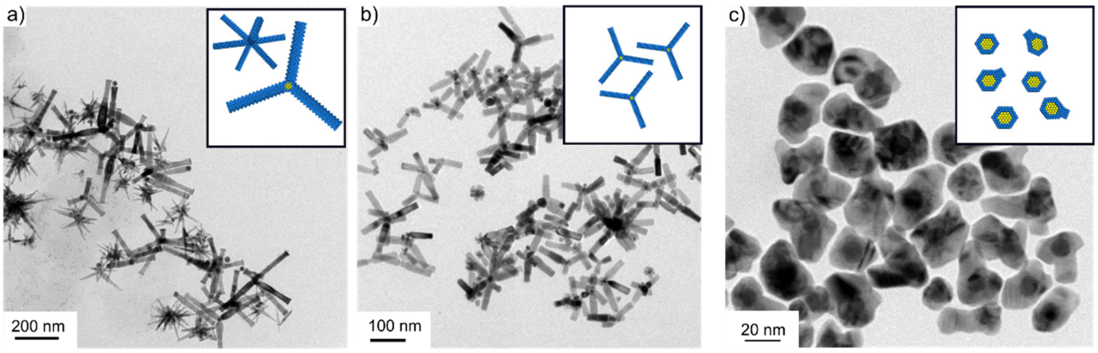


**Figure S13.** TEM images of Ni branches nanoparticles synthesized with different ratio of Ni:Au ratio: **a)** 75:1 **b)** 37.5:1 **c)** 12.5:1 and the corresponding models.

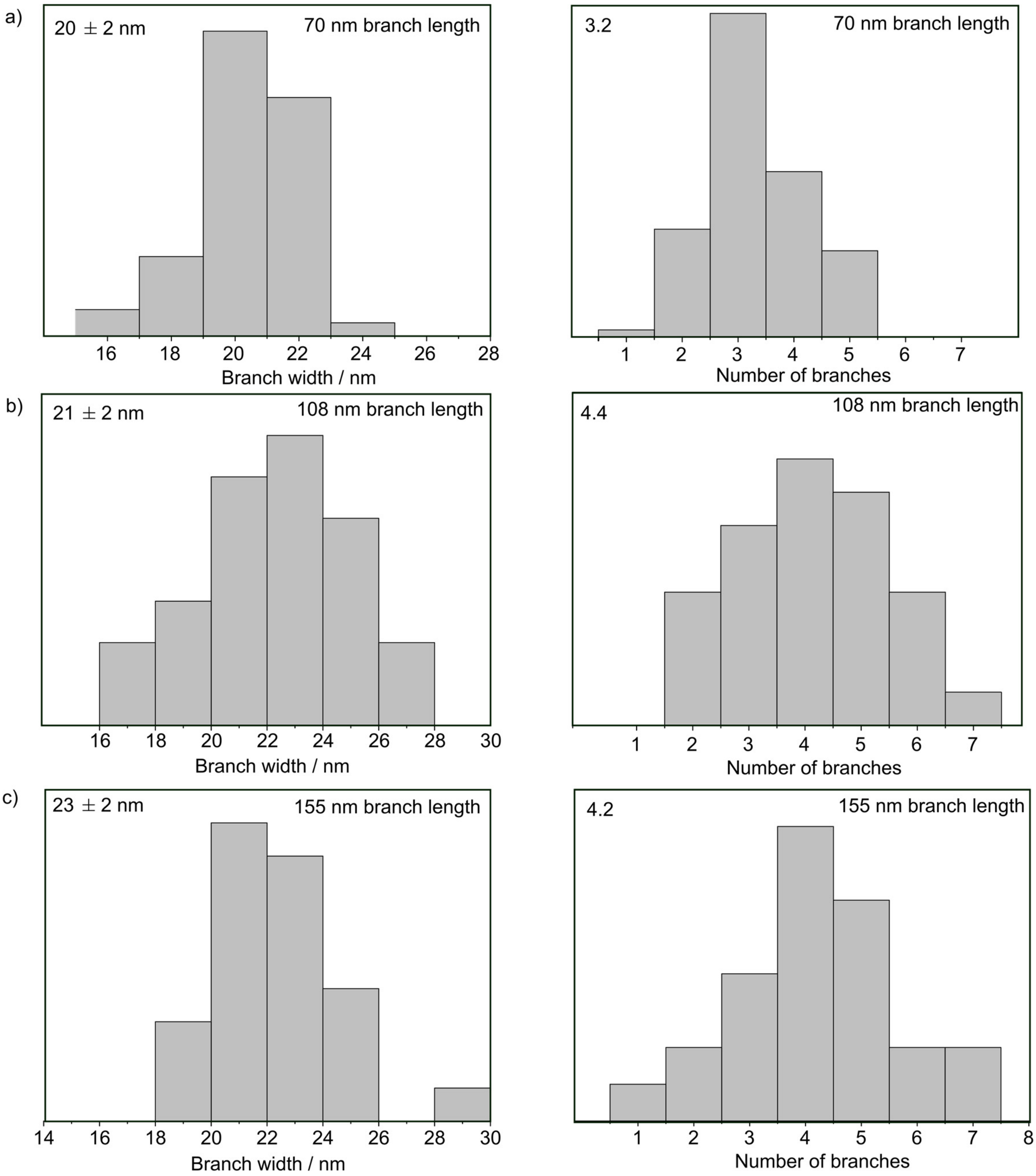


**Figure S14.** Branch width and number of branches analysis for branched Ni nanoparticles with **a)** 70 nm, **b)** 108 nm and **c)** 155 nm branch length. The histograms were obtained from counting 200 nanoparticles.

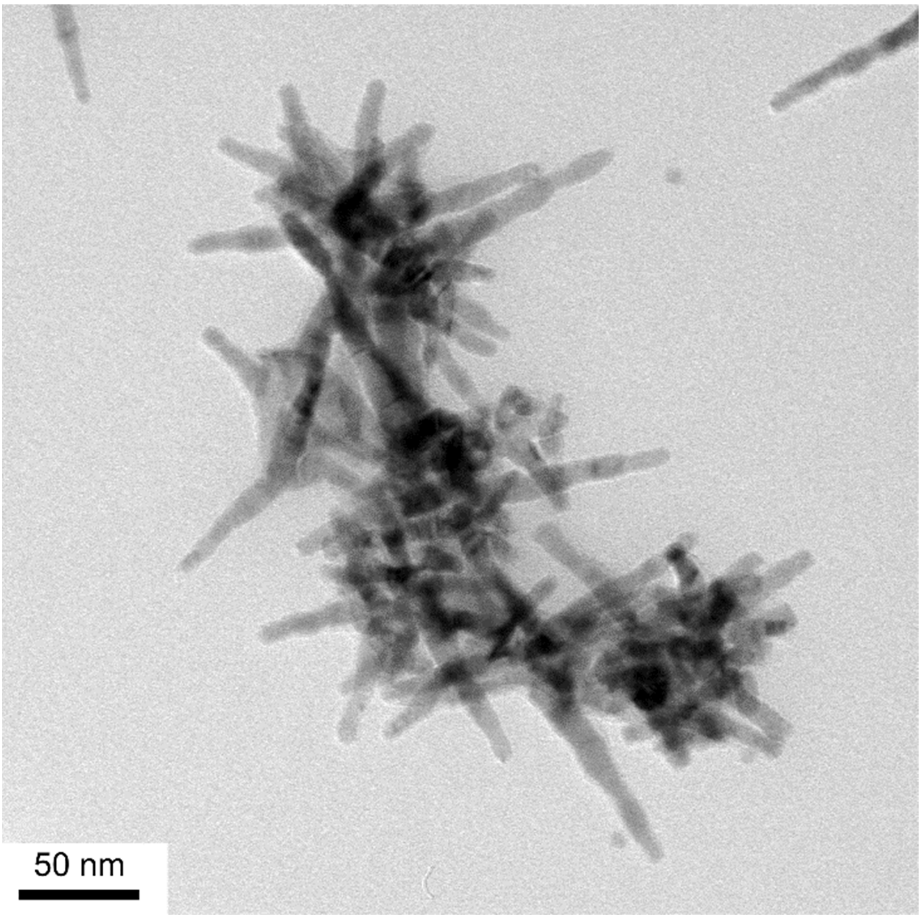


**Figure S15.** TEM image of branched Ni nanoparticles without Au seeds. Other experimental condition was kept identical.

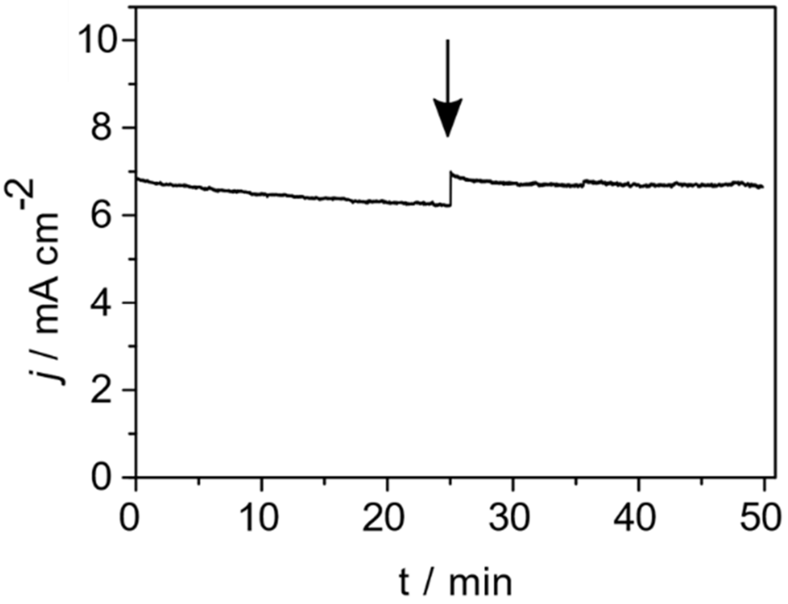


**Figure S16**. Chronoamperometry of branched Ni nanoparticles in 1.0 M KOH and 10 mM HMF. The arrow represents the replacement of the electrolyte. The decrease in current indicates the chemical loss of HMF.

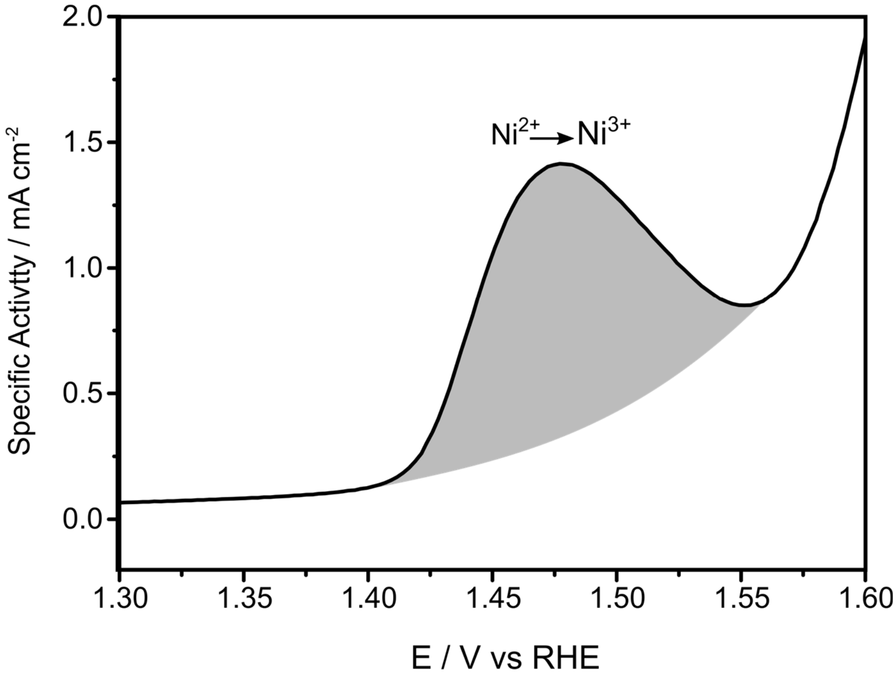


**Figure S17.** LSV responses of branched Ni nanoparticles with 70 nm branch length in 0.1 M KOH. The oxidation peak represents the oxidation of $Ni^{2+}$ to $Ni^{3+}$.

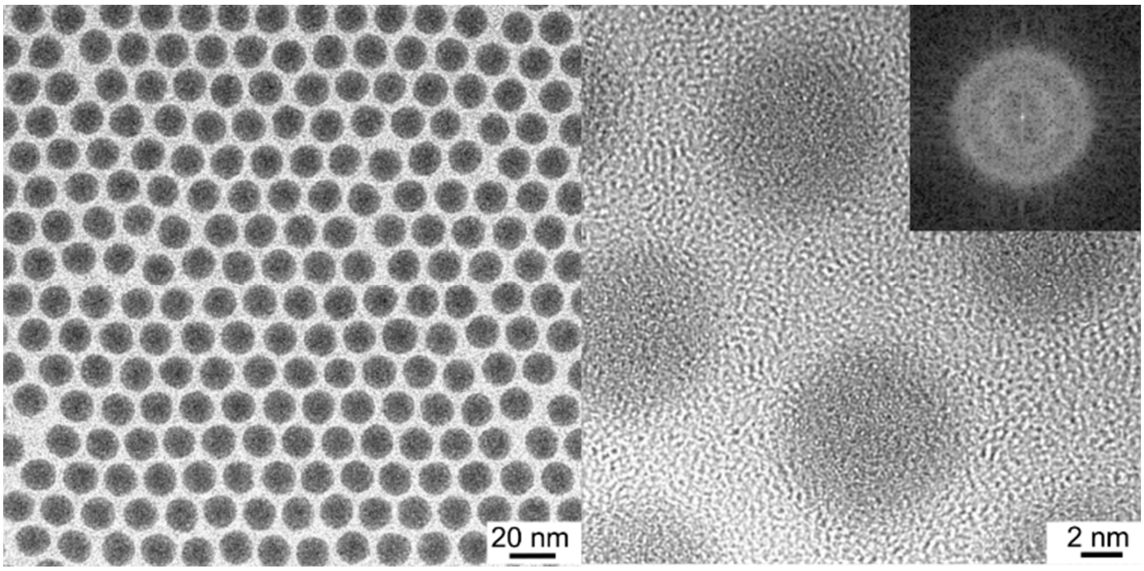


**Figure S18.** TEM and HRTEM images of monodisperse 8 nm Ni nanoparticles used for comparison of the electrocatalytic biomass performance in this work. The FFT shows diffuse which indicates the amorphous structure.

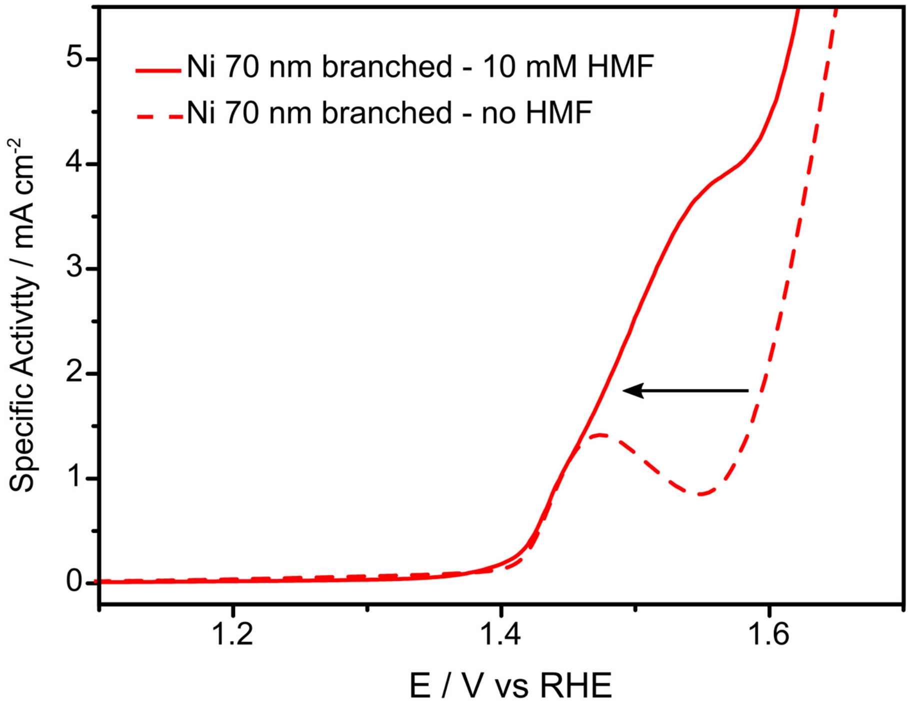


**Figure S19.** LSV curves of branched Ni nanoparticles with 70 nm branch length with (solid line) and without (dash line) 10 mM HMF in 0.1 M KOH. The arrow shows that HMF oxidation requires 130 mV lower potential than OER at 2 mA $cm^{-2}$.

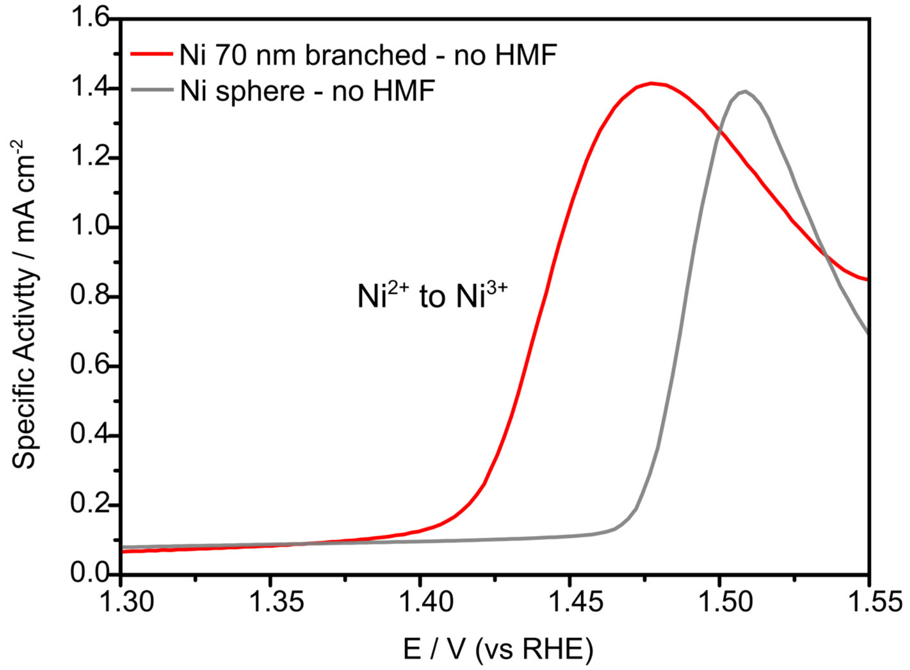


**Figure S20.** LSV curves of Ni 70 nm branched (red line) and sphere Ni nanoparticles (gray line) in 0.1 M KOH without HMF indicating that branched Ni nanoparticles oxidize $Ni^{2+}$ to $Ni^{3+}$ at lower potential.

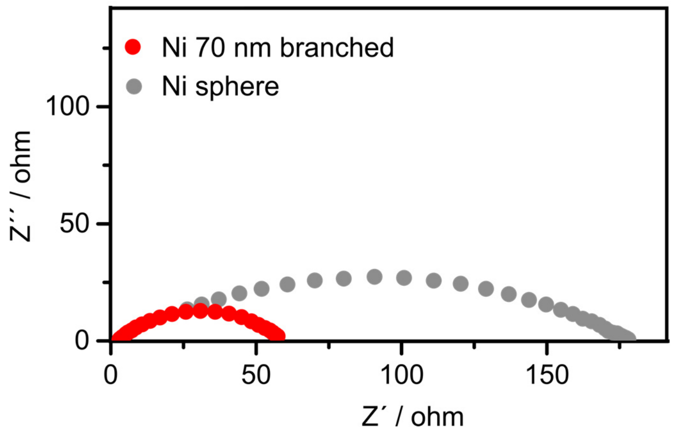


**Figure S21**. EIS of branched and sphere Ni nanoparticles at 1.5 V vs RHE in 0.1 M KOH containing 10 mM HMF.

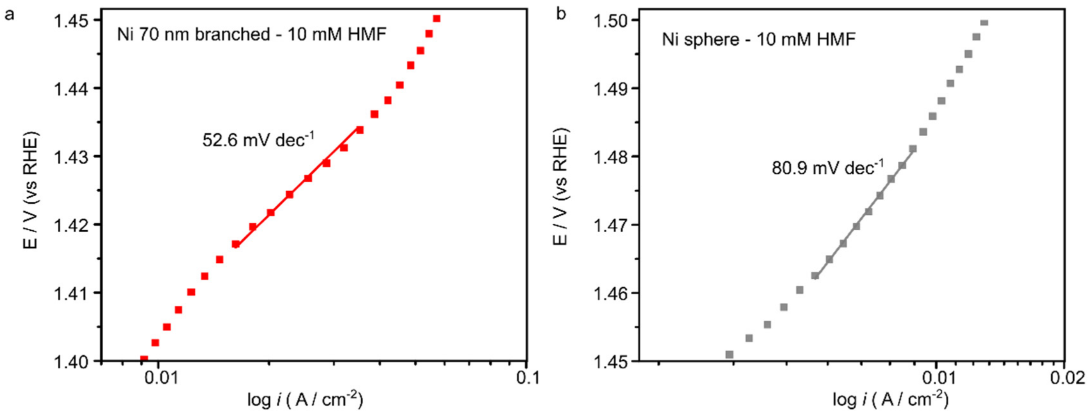


**Figure S22.** Tafel slope of **(a)** Ni 70 nm branched and **(b)** Ni sphere nanoparticles with a scan rate of 5 mV $s^{-1}$ in 0.1 M KOH with 10 mM HMF.

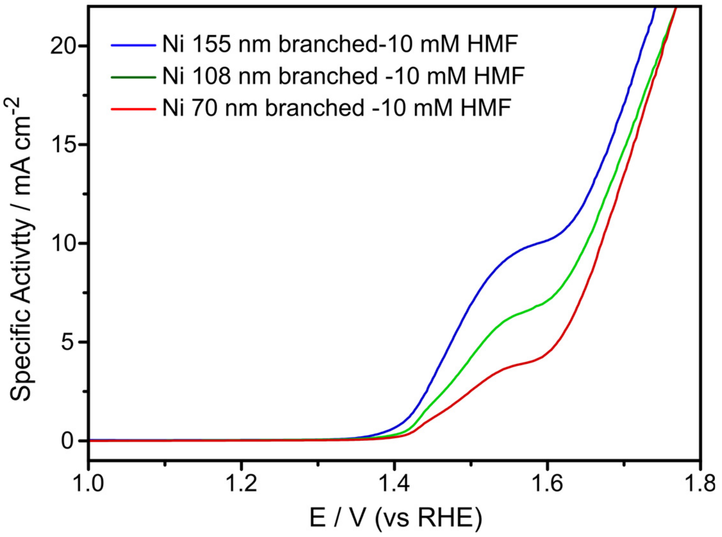


**Figure S23.** LSV curves of branched Ni nanoparticles in 10 mM HMF and 0.1 M KOH with different branch length: 155 nm (blue line), 108 nm (green line) and 70 nm (red line).

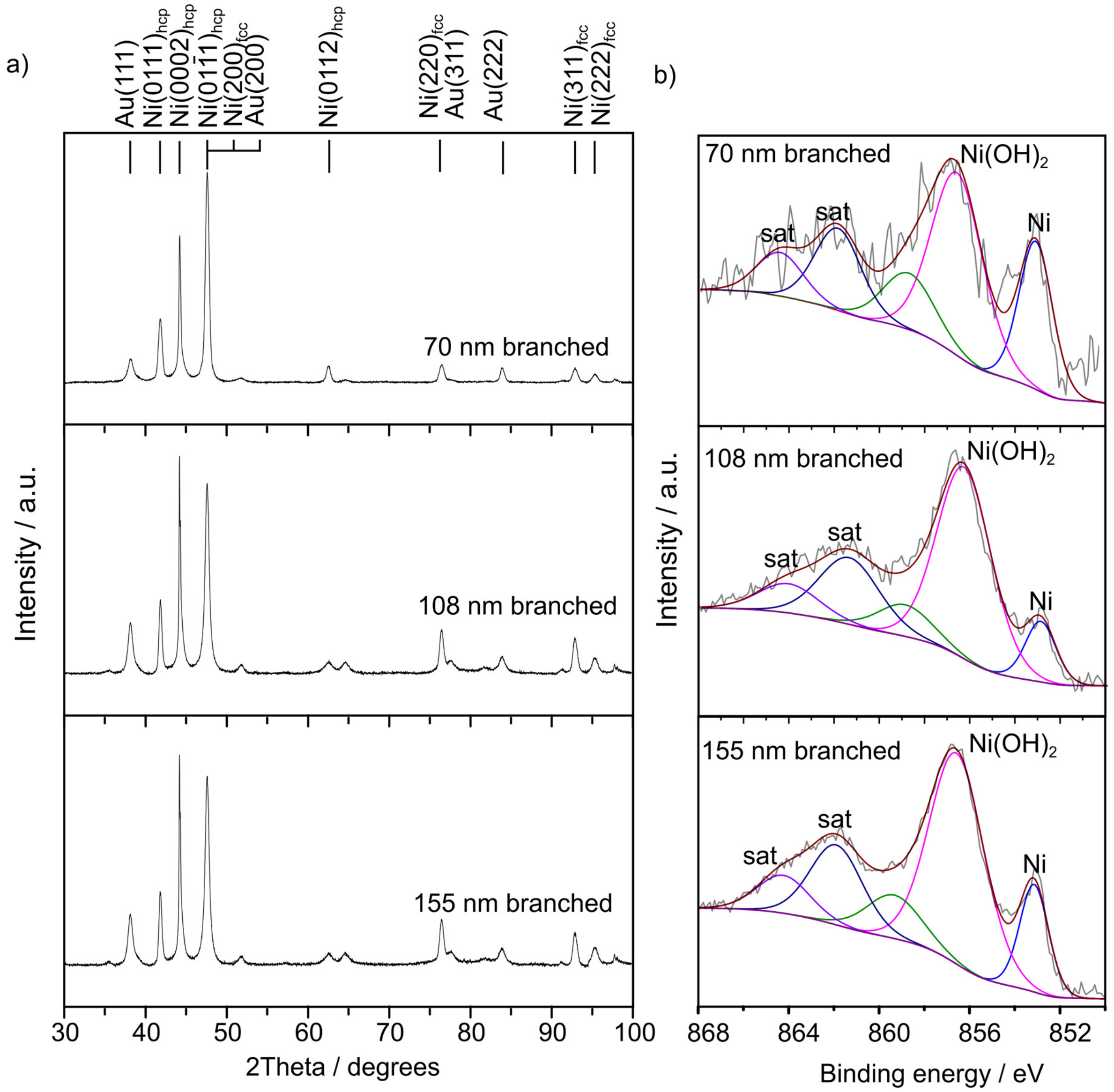


**Figure S24. a)** XRD pattern of Ni branched nanoparticles with different branch length showing the presence of *fcc*-Au, *fcc*-Ni and *hcp*-Ni. **b)** High resolution XPS analysis in the Ni $2p_{3/2}$ region of branched Ni nanoparticle with different branch length showing that all samples have two main Ni species: $Ni^0$ and $Ni^{2+}$. The information depth for XPS is approximately 5 nm, therefore the XPS result is consistent with EDS result which shows that the branches have 3 nm oxide layer. The XRD and XPS results indicates that all branched nanoparticles have the same crystal structure and surface oxidation state.

## Calculation of facet ratio

The facet ratio is calculated by number of atoms on the facets.[2]

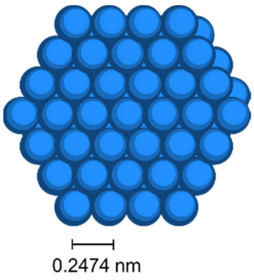


The number of atoms on {0001} facet can be calculated through equations S.1-S.2

$$N_{A\{0001\}} = (n-1)(3n-2)+3n-1 \quad (S.1)$$

$$n_{side} = \frac{d_{side}}{0.247} \quad (S.2)$$

$n_{side}$= number of atoms on the side
$N_{A\{0001\}}$= number of atoms on {0001} facet
$d_{side}$= distance of the side of hexagonal shape

For nanoparticles with 70 nm branch length, $d_{side}$ is measured from TEM images:

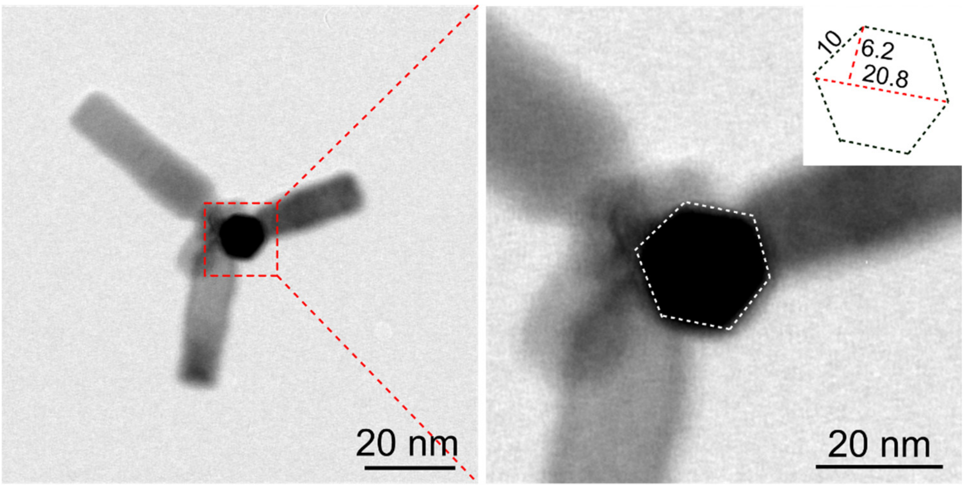


The number of atoms on the side (n) is approximately 40 atoms.

$N_{A\{0001\}}$= 4681 atoms

The branches are enclosed by 6 faces of {10-10} facet.

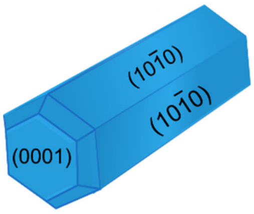


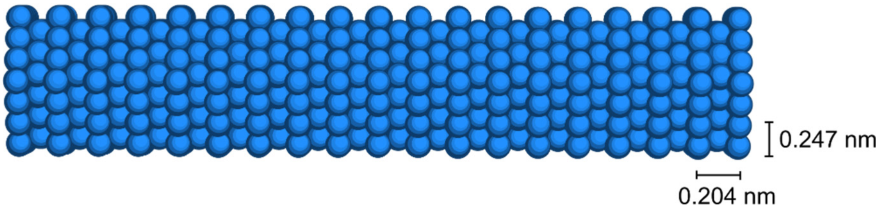


$$N_{A\{10\text{-}10\}} = 6.\, n_{length}.n_{side} \quad (S.3)$$

$$n_{length} = \frac{d_{length}}{0.204} \quad (S.24)$$

$n_{length}$= number of atoms along *c*-axis
$N_{A\{0001\}}$= number of atoms on {0001} facet

For nanoparticles with 70 nm branch length, $n_{side}$ has been calculated above and $n_{length}$ is approximately 350 atoms.

$N_{A\{10\text{-}10\}}$= 6x350x40 = 84000 atoms

Therefore, the facet ratio:

$$facet\ ratio = \frac{N_{A\,\{10-10\}}}{N_{A\,\{0001\}}}$$

**Table S1**. Calculation of the number of atoms and facet ratio.

| Branch length (nm) | Branch width (nm) | $N_A$ on {10-10} facet | $N_A$ on {0001} facet | Ratio of {10-10} to {0001} facets |
|---|---|---|---|---|
| 70 | 20 | 84000 | 4681 | 18 |
| 108 | 21 | 134064 | 5209 | 25 |
| 155 | 23 | 231900 | 6257 | 37 |
| | | | | |

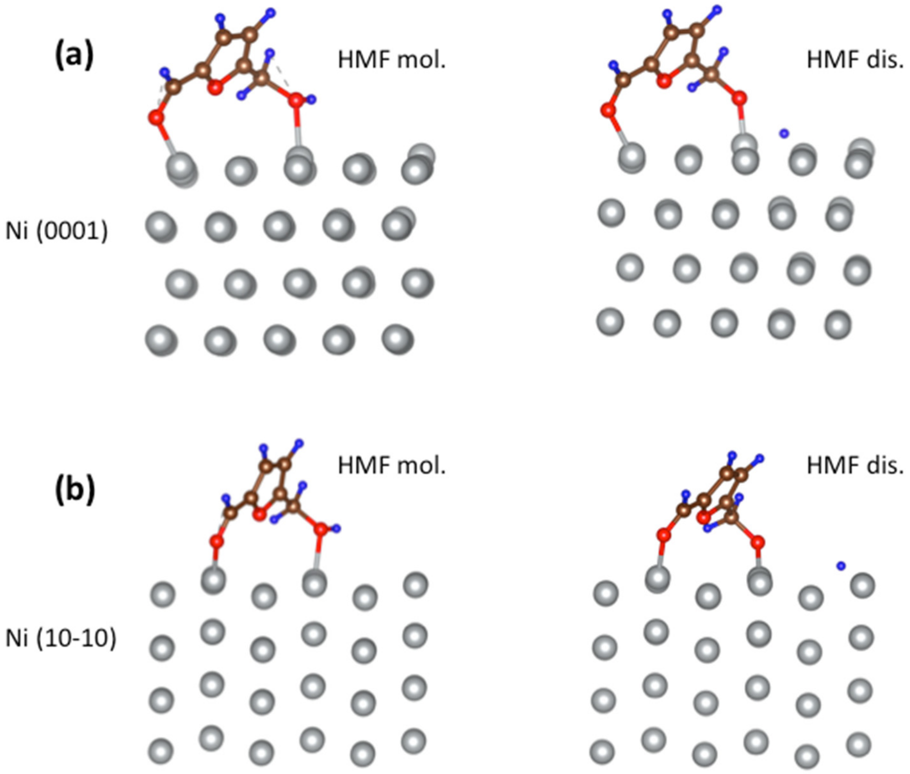


**Figure S25.** Structural models used to model O-H bond dissociation reaction during HMF oxidation on different Ni facets. Grey, brown, red and blue spheres represent Ni, C, O and H atoms, respectively. Mol. and dis. represent molecular and dissociated states, respectively.

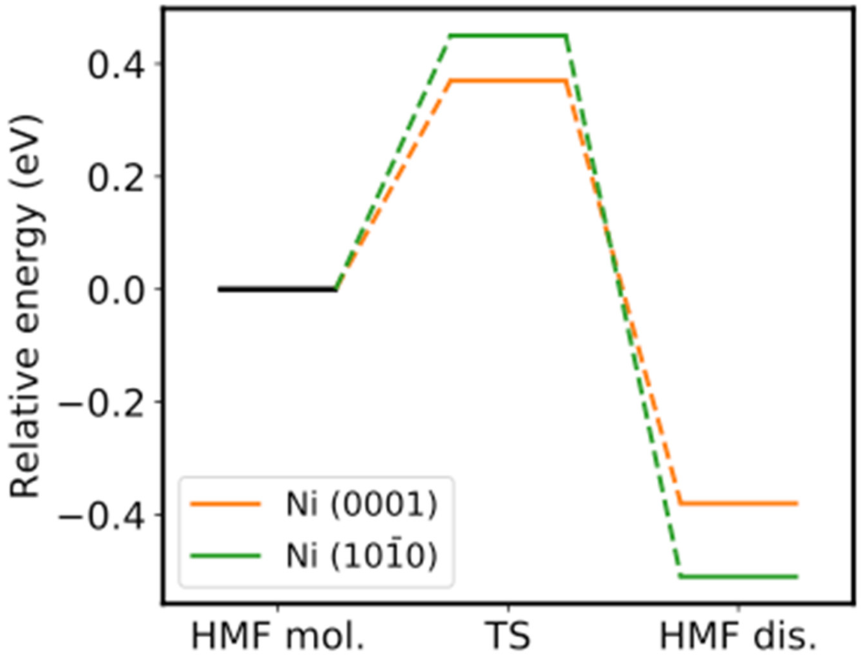


**Figure S26.** Potential energy diagram for O-H bond dissociation reaction on Ni (0001) and (10-10) surfaces. TS is the transition state.

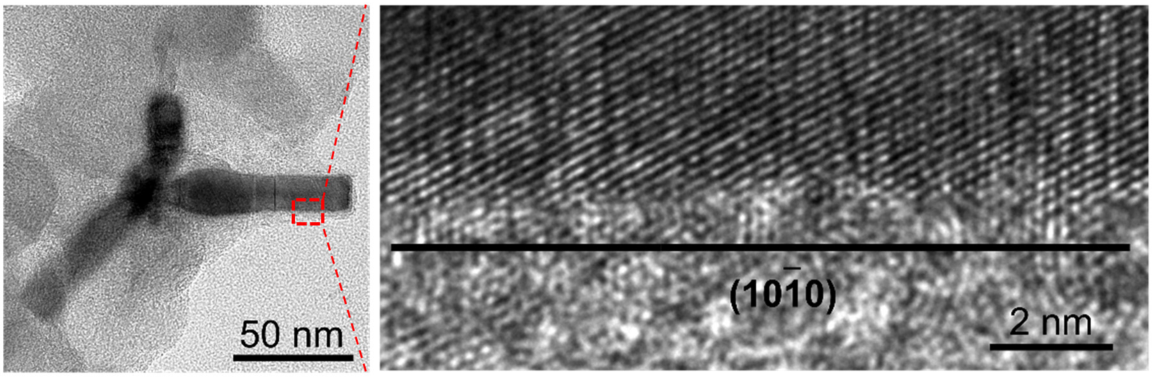


**Figure S27**. TEM image and HRTEM image of branched Ni nanoparticles performed after one hour chronoamperometry at 1.5 V showing that the morphology and surface faceting is stable at high oxidation potentials.

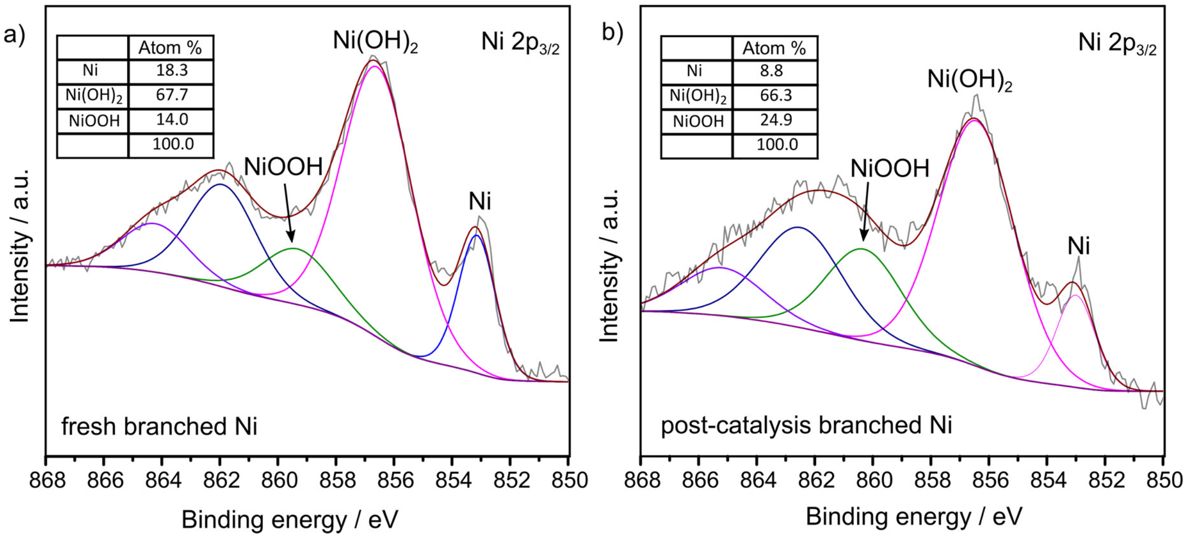


**Figure S28.** XPS spectra in the Ni 2p region of branched Ni nanoparticles. **a)** fresh and **b)** after one hour of chronoamperometry at 1.5 V vs RHE. After catalysis, the proportion of NiOOH increases relatives to Ni and $Ni(OH)_2$ as $Ni^{3+}$ is the main active site for HMF oxidation.

**Table S2.** Comparison of HMF oxidation activity in alkaline solution.

| **Catalysts** | **Electrolyte** | **ECSAs on electrode ($cm^{-2}$)** | **mass loading ($mg/cm^{-2}$)** | **Specific activity (mA $cm^{-2}$) at 1.53 V** | **Mass activity (mA $mg^{-1}$) at 1.45 V** | **Ref** |
|---|---|---|---|---|---|---|
| Faceted branched Ni – 70 nm | 0.1 M KOH+10 mM HMF | 0.54 | 0.1 | 3.3 | 4.6 | this work |
| Faceted branched Ni– 108 nm | 0.1 M KOH+10 mM HMF | 0.46 | 0.1 | 5.7 | 5.6 | this work |
| Faceted branched Ni– 155 nm | 0.1 M KOH+10 mM HMF | 0.33 | 0.1 | 8.5 | 16.8 | this work |
| Sphere Ni | 0.1 M KOH+10 mM HMF | 0.56 | 0.12 | 0.8 | 0.7 | this work |
| $NiCo_2O_4$ foam | 1 M KOH + 5 mM HMF | 6.75 | NA | 2.2 | NA | [10] |
| NiB/Ni foam | 1 M KOH + 10 mM HMF | NA | 1 | NA | 100 | [11] |
| $Ni_3N$@C foam | 1 M KOH + 10 mM HMF | NA | NA | NA | NA | [12] |

NA = not available

## References


[1] M. B. Stevens, L. J. Enman, A. S. Batchellor, M. R. Cosby, A. E. Vise, C. D. M. Trang, S. W. Boettcher, *Chem. Mater.* **2017**, 29, 120-140.

[2] C. M. Zalitis, A. R. Kucernak, J. Sharman, E. Wright, *J. Mater. Chem. A* **2017**, *5*, 23328-23338.

[3] G. Kresse, J. Hafner, *Phys. Rev. B* **1994**, *49*, 14251.

[4] G. Kresse, J. Furthmuller, *Phys. Rev. B* **1996**, *54*, 11169.

[5] G. Kresse, D. Joubert, *Phys. Rev. B* **1999**, *59*, 1758.

[6] J. P. Perdew, K. Burke, M. Ernzerhof, *Phys. Rev. Lett.* **1996**, *77*, 3865.

[7] A. P. LaGrow, B. Ingham, S. Cheong, G. V. M. Williams, C. Dotzler, M. F. Toney, D. A. Jefferson, E. C. Corbos, P. T. Bishop, J. Cookson, R. D. Tilley, *J. Am. Chem. Soc.* **2012**, *134*, 855-858.

[8] A. P. LaGrow, S. Cheong, J. Watt, B. Ingham, M. F. Toney, D. A. Jefferson, R. D. Tilley, *Adv. Mater.* **2013**, 25, 1552-1556.

[9] G. L. Drisko, C. Gatel, P.-F. Fazzini, A. Ibarra, S. Mourdikoudis, V. Bley, K. Fajerwerg, P. Fau, M. Kahn, *Nano Lett.* **2018**, 18, 1733-1738

[10] M. J. Kang, H. Park, J. Jegal, S. Y. Hwang, Y. S. Kang, H. G. Cha, *Appl. Catal. B: Environ.* **2019**, *242*, 85-91.

[11] S. Barwe, J. Weidner, S. Cychy, D. M. Morales, S. Dieckhöfer, D. Hiltrop, J. Masa, M. Muhler, W. Schuhmann, *Angew. Chem. Int. Ed.* **2018**, *57*, 11460-11464; *Angew. Chem.* **2018**, 130, 11631-11636

[12] Zhang, Y. Zou, L. Tao, W. Chen, L. Zhou, Z. Liu, B. Zhou, G. Huang, H. Lin, S. Wang, *Angew. Chem. Int. Ed.* **2019**, *58*, 15895-15903; *Angew. Chem.* **2019**, 131, 12841-12846
